# Validation of the Brazilian version of the Short Inventory of Grazing (SIG)

**DOI:** 10.47626/2237-6089-2022-0492

**Published:** 2024-01-08

**Authors:** Carlos Eduardo Ferreira Moraes, Andreea Heriseanu, Carla Mourilhe, Ana Luisa Kremer Faller, Phillipa Hay, Jose Carlos Appolinario

**Affiliations:** 1 Grupo de Obesidade e Transtornos Alimentares Instituto de Psiquiatria Universidade Federal do Rio de Janeiro Rio de Janeiro RJ Brazil Grupo de Obesidade e Transtornos Alimentares (GOTA), Instituto de Psiquiatria , Universidade Federal do Rio de Janeiro (UFRJ), Rio de Janeiro , RJ , Brazil .; 2 Department of Psychology eCentreClinic Macquarie University Sydney NSW Australia Department of Psychology , eCentreClinic , Macquarie University , Sydney , NSW , Australia .; 3 Departamento de Nutrição Social e Aplicada Instituto de Nutrição Josué de Castro UFRJ Rio de Janeiro RJ Brazil Departamento de Nutrição Social e Aplicada , Instituto de Nutrição Josué de Castro , UFRJ , Rio de Janeiro , RJ , Brazil .; 4 Translational Health Research Institute School of Medicine Western Sydney University Sydney NSW Australia Translational Health Research Institute , School of Medicine , Western Sydney University , Sydney , NSW , Australia .

**Keywords:** The Short Inventory of Grazing, validation, Brazil, self-report instruments, eating-related psychopathology

## Abstract

**Objective:**

Grazing is a disturbed eating pattern that has been associated with eating disorders and obesity. One of the new measures to investigate this eating behavior is the Short Inventory of Grazing (SIG), a two-item questionnaire that assesses grazing in general and grazing associated with the feeling of loss of control over eating (LOC grazing). However, the psychometric properties of the SIG have not been assessed in the Brazilian population. The present study aimed to cross-culturally adapt and validate a Brazilian version of the SIG.

**Methods:**

The SIG was adapted to the Brazilian context following international guidelines. Then, 90 undergraduate students completed an online survey including questions from the SIG, the Binge Eating Scale (BES), the Patient Health Questionnaire-9 (PHQ9), the Generalized Anxiety Disorder-7 (GAD7), and a question related to self-reported health status. The internal consistency, test-retest reliability, and convergent validity of the questionnaire were assessed.

**Results:**

The prevalence rates of at least one weekly episode of grazing in general and LOC grazing were 71.1 and 54.5%, respectively. The internal consistence of the SIG was acceptable (0.81). In addition, SIG scores on both items were positively and significantly associated with BES, GAD7, and PHQ9 scores, and with poorer self-rated health. However, SIG test and retest scores differed significantly.

**Conclusion:**

Overall, the Brazilian version of the SIG demonstrated adequate psychometric properties. The instrument had adequate internal consistency, with both items exhibiting good convergent validity with related measures.

## Introduction

Grazing is a disturbed eating behavior characterized by repetitive consumption of small amounts of food over long periods, outside of regular meals or snacks, and without planning. In addition, this unstructured eating is not in response to sensations of hunger or satiety. ^1^ Considering its associations with external and emotional eating, it is hypothesized that grazing could be regarded as a habitual behavior, performed automatically in response to aversive emotional states and exposure to food cues in the environment. ^2 , 3^ Recently, some authors proposed that grazing can be divided into two subtypes: 1) compulsive grazing (CG), in which grazing is associated with the feeling of loss of control (LOC) over eating; and 2) non-compulsive grazing (NCG), characterized by repetitive and distracted eating, without LOC. ^1 , 2 , 4^

Grazing seems to be a common eating disordered behavior in both clinical and non-clinical samples. Heriseanu et al. ^3^ performed a systematic review with metanalysis about grazing prevalence in individuals with obesity and eating disorders (ED). They found a mean pooled prevalence of 33.2% in individuals seeking obesity treatment. Regarding subjects with ED, authors reported the following prevalence rates: 67.7% for binge eating disorder (BED), 58.2% for bulimia nervosa (BN), and 34.3% for anorexia nervosa (AN). ^3^ In non-clinical contexts, grazing occurs at least once a week in more than 80% of undergraduate students and general community samples. ^5 , 6^ In addition, the point prevalence rates of LOC grazing and grazing without LOC are 10.2% and 38%, respectively.

There is some evidence that grazing may impact weight loss treatments, general and eating-related psychopathology, and quality of life. ^3^ In clinical contexts, the presence of grazing negatively impacts weight loss maintenance and weight regain after weight loss treatment. ^3^ In addition, individuals with obesity and grazing display a greater frequency of binge eating episodes, more severe symptoms of depression and anxiety, and lower quality of life. ^3^ In community settings, grazing has been positively correlated with body mass index (BMI), psychological distress, and ED symptomatology (e.g., cognitive restraint, weight, shape, and eating concerns). ^7 , 8^ However, individuals with LOC grazing exhibited higher levels of ED pathology than those with non-compulsive grazing. ^7^

Grazing can be considered an individual attempt to regulate emotional states. ^2^ It can be impacted by stressful and emotionally activating occasions, such as the coronavirus disease 2019 (COVID-19). ^9^ Overall, the lockdown due to COVID-19 affected peoples’ psychosocial functioning. ^10 , 11^ Consequently, during this period there was an increase in stress, anxiety, depression, and disordered eating behaviors such as grazing and binge eating episodes. ^9 - 13^ Regarding grazing, a community-based study conducted by Ramalho et al. ^13^ revealed a prevalence of 80.9% during the first mandatory lockdown, in Portugal. These authors indicated that changes in the daily routine during the pandemic led to increased psychological distress and resulted in more disordered eating. ^13^ Taken together, these findings suggest that the coronavirus outbreak negatively impacted eating behaviors and mental health.

Despite the growing interest in studying grazing, there are few instruments specifically developed to assess this eating behavior. The Grazing Questionnaire is a seven-item instrument that assesses grazing severity considering the time spent on grazing episodes. ^2^ However, it does not provide information about grazing frequency. The Rep(eat)-Q is a 12-item questionnaire that evaluates grazing frequency in the previous 4 weeks. ^8^ Nevertheless, this is a relatively short time frame to assess whether an eating behavior occurs regularly. Also, the Rep(eat)-Q can be time-consuming to employ in epidemiological surveys due to the number of items. To overcome the limitations of the previous measures, Heriseanu et al. developed the Short Inventory of Grazing (SIG). ^4^ This is a two-item questionnaire that evaluates the frequency and severity of grazing in general and LOC grazing. ^4^ However, its psychometric properties have not been assessed in the Brazilian population. Thus, the present study aimed to cross-culturally adapt and validate a Brazilian version of the SIG.

## Methods

### Participants and procedures

A sample of 90 undergraduate students enrolled on the dietitians’ course at the Universidade Federal do Rio de Janeiro (UFRJ) was invited to participate in this study through e-mail and social media advertisements explaining study’s aims and procedures. They were also sent a link to access an online form containing questions about sociodemographic and clinical information, health status, and general and eating-related psychopathology. Data collection was performed between May and September 2021. This research was approved by the ethics committee at the Instituto de Psiquiatria, UFRJ. Online informed consent was obtained from all study participants before any procedures were performed.

To assess the temporal stability of the SIG, participants who completed the survey questionnaire were invited to answer the questions about grazing again within a 2-week interval. This period is considered sufficient to avoid temporal changes in the answers. ^14^ The assessments were independent and participants did not have access to the results of the first evaluation.

### Measures

#### Short Inventory of Grazing (SIG)

The SIG is a two-item questionnaire developed to independently assess the frequency and severity of grazing in general (first item) and LOC grazing (second item). Grazing frequency is rated on a seven-point scale ranging from “none at all” to “eight or more times a week.” ^4^ The SIG does not provide a cut-off point based on a dimensional scale. Presence of regular grazing episodes is defined as grazing at least once a week in the previous 3 months. ^4^ For the assessment of grazing severity, episodes are categorized according to their frequency similarly to the Diagnostic and Statistical Manual of Mental Disorders, 5th edition (DSM-5) ^15^ criteria for BED severity, as follows: mild (one-three episodes per week), moderate (four-seven episodes per week), or severe (eight or more episodes per week). ^4^ As “grazing in general” encompasses grazing with and without LOC, Heriseanu et al. ^5^ proposed establishing the following two mutually exclusive categories of grazing according to SIG scores:

- Grazing without LOC: comprises individuals who endorsed regular episodes of grazing without LOC but did not engage in regular LOC grazing.
- LOC grazing: comprises individuals who engaged in regular episodes of grazing accompanied by the feeling of LOC over-eating.

Since the categories are mutually exclusive, participants who endorsed both types of grazing are categorized as engaging only in LOC grazing.

Permission to cross-culturally adapt the SIG for the Brazilian context was requested from and granted by the authors of the original version of the questionnaire. The translation process was performed through the following steps ^16 , 17^ : 1) two independent forward translations were performed by bilingual researchers experienced in the field of ED; 2) ambiguities and discrepancies in the two translations were discussed by a committee of 10 ED specialists; 3) a blind back-translation to English was performed by a bilingual person; 4) items were discussed with one of the authors of the original SIG; 5) the final version was approved by the investigators and is available at online-only supplementary material.

#### Binge Eating Scale (BES)

The BES is a 16-item questionnaire developed to assess the presence and severity of binge eating symptoms. ^18^ Each item presents a range of three to four statements regarding an aspect of binge eating (e.g., “I can control my impulses towards food” to “I feel totally unable to control my relationship with food and I try desperately to fight my impulses toward food”). BES scores vary from 0 to 46 points. Scores between 18 and 26 suggest the presence of moderate binge eating. ^18^ Values greater than 26 indicate severe binge eating. ^18^ The BES was translated to Portuguese and validated for the Brazilian context in a sample of women with obesity. ^19 , 20^ The questionnaire was considered a valid measure for screening of BED (sensitivity: 97.8%; specificity: 47.7%; positive predictive value: 66.7%; negative predictive value: 95.3%). ^20^ Cronbach’s alpha for this study sample was 0.89.

#### Patient Health Questionnaire 9 (PHQ9)

The PHQ9 is a nine-item questionnaire that assesses the presence of symptoms of depression in the previous 2 weeks, according to Diagnostic and Statistical Manual of Mental Disorders, 4th edition (DSM-IV) ^21 , 22^ criteria (e.g., “over the last 2 weeks, how often have you been bothered by little interest or pleasure in doing things?”). The frequency of symptoms is evaluated on a four-point scale ranging from “none at all” to “almost every day.” ^21^ The Brazilian version of the PHQ9 was validated in a population-based study and a cut-off point of ≥ 9 showed the highest sensitivity (77.5%) and specificity (86.7%) for screening for major depressive disorder. ^23^ In the present study, the PHQ-9 exhibited a Cronbach’s alpha of 0.88.

#### Generalized Anxiety Disorder 7 (GAD7)

The GAD-7 is a seven-item scale developed to measure generalized anxiety symptoms ^24^ (e.g., “over the last 2 weeks, how often have you been bothered by feeling nervous, anxious or on edge?”). Each item is scored on a four-point scale based on the frequency of the symptoms over the last 2 weeks (e.g.: “not at all”; “several days”). ^24^ Total scores range from 0 to 21 with higher values indicating higher symptomatology. ^24^ The GAD-7 was translated to Brazilian Portuguese and validated in a community sample of adults. ^25^ Cronbach’s alpha for this study sample was 0.89.

#### Self-rated health status

Health status was assessed using a question from the 12-Item Short-Form Health Survey (SF-12). ^26^ This is a self-report instrument comprising questions to evaluate components of physical and mental health. ^26^ The SF-12 is widely used for assessment of health-related quality of life. ^26^ The Brazilian Portuguese version of the SF-12 was validated in a non-clinical sample and showed good psychometric properties (convergent validity and reliability). ^27^ For this study, the first item of the questionnaire was employed, as follows: “In general, would you say your health is:”. This item is rated on a five-point scale (1 = “excellent”, 2 = “very good”, 3 = “good”, 4 = “fair”, and 5 = “poor”). ^26^

## Sociodemographic and anthropometric information

The following sociodemographic characteristics were assessed: age (years), sex (male, female), and ethnicity (white; non-white [including black, mixed, oriental, and indigenous]). Anthropometric information included self-reported weight (kg) and height (m). In addition, BMI was calculated (BMI = weight/height ^2^ in kg/m ^2^ ) and categorized as follows: underweight (< 18.5); normal weight (18.5-24.9); overweight (25.0-29.9); and obesity (≥ 30). ^28^

## Statistical analysis

Data were inspected for normality using the Kolmogorov-Smirnov test. This is the recommended method for testing the normality of data in samples greater than 50. ^29^ Analysis revealed that age, BMI, and GAD-7, PHQ-9, and BES scores were not normally distributed (p < 0.001). Non-parametric tests were therefore employed. The sample was characterized in terms of sociodemographic variables, BMI, and scores on the measures of general and eating-related psychopathology (frequencies, means, standard deviations, minimum and maximum values). The Spearman-Brown coefficient was employed to assess the internal consistency of the SIG. This coefficient was calculated for the correlation between the two items of the questionnaire. This is the preferred method for assessment of internal consistency of two-item measures because it is less biased by the number of questionnaire items than Cronbach’s alpha. ^30^ Test-retest reliability was also assessed using Wilcoxon’s signed-rank test and Kendall’s tau-b.

Correlations between the SIG and related measures were calculated using Kendall’s tau-b association (for non-normally distributed data) and effect sizes were based on Cohen’s guidelines for r. As Kendall’s tau-b is not directly interpretable and yields smaller values than r, a conversion was employed. ^31 , 32^ Thus, the following cut-offs for effect size were used: small (tau = 0.06 [equivalent to r = 0.1]); medium (tau = 0.19 [equivalent to r = 0.3]); and large (tau = 0.33 [equivalent to r = 0.5]).

Further analyses were performed to compare participants who engaged in regular LOC grazing against those with regular episodes of grazing without LOC in terms of demographic variables, BMI, and general and eating-related psychopathology. Between-group differences were analyzed with Kruskal-Wallis and chi-square tests. The following cut-offs for effect size were used: eta ^2^ = 0.01 ≤ 0.06 (small); eta ^2^ = 0.06 ≤ 0.14 (medium); and eta ^2^ ≤ 0.14 (large). ^33^

Statistical analyses were conducted using the Statistical Package for the Social Sciences (SPSS), version 22. Statistical significance was set at p < 0.05.

## Results

### Sample characteristics

Table 1 lists the demographic and anthropometric characteristics of the sample, the scores obtained from the study measures of general and eating-related psychopathology, and self-rated health status.

**Table 1:** Participants’ sociodemographic and anthropometric information, scores obtained from the study measures of general and eating-related psychopathology, and health status

Variables	n = 90	Min-Max
Age, mean (SD)	22.4 (3.8)	18-38
Sex, n (%)		
Female	85 (94.4)	NA
Male	5 (5.6)	NA
Ethnicity, n (%)		
White	57 (63.3)	NA
Non-white	33 (36.7)	NA
BMI, mean (SD)	23.2 (3.8)	17.3-37.8
BMI category, n (%)		
Low weight	9 (10.0)	NA
Normal weight	54 (60.0)	NA
Overweight	23 (25.6)	NA
Obesity	4 (4.4)	NA
BES score, mean (SD)	9.0 (7.4)	0-34
GAD7 score, mean (SD)	8.7 (5.3)	0-21
PHQ9 score, mean (SD)	9.9 (6.4)	0-24
Self-rated health, n (%)		
Excellent	4 (4.4)	NA
Very good	26 (28.9)	NA
Good	51 (56.7)	NA
Bad	9 (10.0)	NA

BES = Binge Eating Scale; BMI = body mass index; GAD7 = Generalized Anxiety Disorder-7; NA = not applicable; PHQ9 = Patient Health Questionnaire-9; SD = standard deviation.

### Grazing frequency

The prevalence of grazing in general was 71.1%. In this regard, most of the participants reported “mild” or “moderate” grazing (54.4%). The frequency of regular LOC grazing (≥ 1 episode/week) was 51.1%, with most of the subjects endorsing “mild” LOC grazing (36.7%) ( Table 2 ).

**Table 2:** Frequency of grazing

Frequency of grazing (episodes/week)	SIG 1 – Grazing in general n (%)	SIG 2 – Grazing with LOC* n (%)
No grazing/< 1	26 (28.9)	44 (48.9)
1-3 (mild)	25 (27.8)	33 (36.7)
4-7 (moderate)	24 (26.7)	7 (7.8)
8 or more (severe)	15 (16.7)	6 (6.7)

LOC = loss of control; SIG = Short Inventory of Grazing.

* Grazing in general includes grazing with and without LOC.

### Psychometric properties of SIG

#### Internal consistency

The results indicate that the Brazilian version of the SIG had a Spearman-Brown coefficient of 0.81. In addition, the two SIG items were strongly associated with each other (Kendall’s tau-b = 0.553; p < 0.001).

#### Test-retest reliability

Participants were asked to answer the SIG again within an interval of 2 weeks. A total of 44 (48.8%) subjects completed the second administration of the SIG (retest). The Wilcoxon signed-rank test revealed that scores for both SIG items showed a statistically significant difference between test and retest (Grazing in general: Z = -2.909, p = 0.004; LOC grazing: Z = -3.637, p < 0.001). Associations between test and retest were statistically significant for the LOC grazing item (Kendall’s tau-b = 0.324; p = 0.03). Conversely, associations between the first and the second applications of the SIG were not statistically significant for the grazing in general item (Kendall’s tau-b = -0.162; p = 0.17).

#### Associations between the SIG and related measures

The associations between scores on both SIG items and other study measures were estimated. Grazing in general and LOC grazing were significantly and positively associated with BES, GAD-7, and PHQ-9 scores (p < 0.001). In addition, both items were significantly and positively associated with poorer self-rated health (p = 0.05) ( Table 3 ).

**Table 3:** Associations between SIG items and demographic characteristics and clinical and psychological variables

Variables	SIG 1 – Grazing in general	SIG 2 – Grazing with LOC
Age	0.073	0.062
Sex	-0.008	-0.116
BMI	0.142	0.067
BES score	0.409*	0.314*
GAD7 score	0.273*	0.302*
PHQ9 score	0.312*	0.297*
Poor self-rated health	**0.208** ^†^	**0.222** ^†^

BES = Binge Eating Scale; BMI = body mass index; GAD7 = Generalized Anxiety Disorder-7;

PHQ9 = Patient Health Questionnaire-9; SIG = Short Inventory of Grazing.

Associations shown are Kendall tau-b values.

Bold figures indicate significant associations with a medium or larger effect size.

* p < 0.01; ^†^ p < 0.05.

## Comparisons between grazing with and without LOC

We performed additional analyses to compare differences regarding sex, age, BMI, psychological aspects, and binge eating symptomatology between participants with regular LOC grazing (n = 46) and those who engaged only in grazing without LOC (n = 22). As both groups were mostly composed of women, no statistical sex difference was found (χ ^2^_[1]_ =1.688, p = 0.24). Similarly, self-rated health did not differ between participants with LOC grazing and those who engaged in grazing without LOC (χ ^2^_[3]_ = 4.583, p = 0.20). The Kruskal-Wallis test revealed that individuals with LOC grazing showed significantly greater depression (H[1] = 3.429, p = 0.05) and anxiety symptoms (H[1] = 5.352, p = 0.02). Although subjects with LOC grazing exhibited greater binge eating symptomatology than those engaging only in grazing without LOC, these differences were not statistically significant (H[1] = 2.068, p = 0.15) ( Table 4 ).

**Table 4:** Differences in psychological and eating-related psychopathology scores between participants with and without LOC grazing

Variables	Grazing without LOC (n=22) Mean (Min; Max)	Grazing with LOC (n=46) Mean (Min; Max)	χ ^2^ /H	p-value
Age (years)	22.4 (19; 36)	22.7 (19; 38)	0.08	0.78
BMI (kg/m ^2^ )	23.7 (17.6; 30.8)	23.4 (17.3; 37.8)	0.12	0.73
BES score	7.8 (0; 17)	11.6 (1; 34)	2.07	0.15
GAD7 score	7.6 (0; 21)	10.5 (1; 20)	**5.35**	**0.02**
PHQ9 score	9.0 (0; 20)	11.9 (2; 23)	3.43	0.05

BES = Binge Eating Scale; BMI = body mass index; GAD7 = Generalized Anxiety Disorder-7;

LOC = loss of control; PHQ9 = Patient Health Questionnaire-9.

Bold figures indicate statistically significant differences with medium or larger effect size.

## Discussion

In the present study, we conducted cross-cultural adaptation of the SIG into Brazilian Portuguese and assessed its psychometric properties in a sample of undergraduate students. To the best of our knowledge, this is the first translation and validation of a self-report instrument that assesses grazing for the Brazilian context. The SIG was adapted following standardized steps according to guidelines for cross-cultural adaptation. Overall, the Brazilian version of SIG showed satisfactory psychometric properties, such as adequate internal consistency and positive associations with related constructs. However, the questionnaire exhibited low stability over the time when applied twice within a 2-week interval.

Overall, our results are similar to those found by Heriseanu et al. ^4^ in the validation study for the original version of the SIG. They assessed the psychometric properties of the questionnaire in a non-clinical sample of both university students and subjects from the general community. They reported a Spearman-Brown coefficient of 0.73, with a strong association between the two items. In addition, both grazing in general and LOC grazing items were positively associated with measures of eating-related psychopathology, such as binge eating, weight and shape concerns, and LOC eating. ^4^

Although the greater part of our results were in line with the literature, there are some findings that were divergent from the previous research. We found that the scores for both SIG items differed significantly between the test and the retest. This suggests that the diagnosis of grazing was not stable over time in our sample. Conversely, the original versions of the SIG (LOC grazing item) and Rep(eat)-Q showed good test-retest reliability after two applications within intervals of 1 and 2 weeks, respectively. ^4 , 8^ Additionally, both of those studies reported statistically significant associations between grazing (specially LOC grazing) and BMI. ^4 , 8^ In the present study, although we did find positive associations between SIG items and BMI, they were not statistically significant.

In the current study, individuals who engaged in regular episodes of grazing associated with LOC over-eating showed greater impact on general and eating-related psychopathology than those with regular episodes of grazing without LOC. Similarly, Conceição et al. ^8^ found that LOC grazing was strongly associated with ED psychopathology in both clinical and non-clinical samples. Accordingly, Heriseanu et al. ^4^ reported that subjects with LOC grazing showed greater general psychopathology, ED, and binge eating symptomatology than those with grazing without LOC. Taken together, these findings support the idea that LOC grazing seems to be a distinct category of grazing. However, further research is required to better understand the role of LOC over-eating in the psychopathology of grazing.

Our findings suggest that the Brazilian version of the SIG showed significant associations with related constructs, such as general and eating psychopathology measures. However, SIG items showed low test-retest reliability and were not significantly associated with BMI. Potential explanations for these findings include the following:

1. The temporal stability of an instrument may be influenced by the test conditions. ^17^ As our study was an online survey, we could not guarantee that both test and retest were performed in similar settings.
2. The small sample size of the present study. Usually, a minimum of 50 subjects is recommended for reliability studies. ^34 , 35^ Although 90 participants completed the first assessment (test), only 44 participated in the retest. Thus, this would have underestimated the temporal stability of SIG.
3. Our sample was composed of students from a dietitians’ course. They have an increased knowledge about food and nutrition, which helps them maintain their BMI within the healthy range. ^36 , 37^ Therefore, the low prevalence of underweight and obesity may have impacted the associations between grazing and BMI.

This study has some limitations. First, the study sample was somewhat homogeneous, as it was comprised predominantly of young women on an undergraduate dietitians’ course and with BMI in the normal range. Thus, this limits generalization of the findings to different contexts. Second, the lack of a sample size calculation could have impacted the accuracy of the SIG. However, Sousa et al. ^16^ recommend at least 10 participants per item on a questionnaire as the minimum sample size for validation studies. Third, only 44 of the 90 participants in the study completed the second administration of the SIG. The low retest response rate (49%) may have negatively influenced the temporal stability of the SIG. Fourth, the use of self-reported weight and height to assess BMI. Although there is a risk of recall bias, such measures have been validated in clinical and non-clinical samples. ^38 , 39^ Despite these limitations, our study also has strengths: 1) the SIG was translated into Brazilian Portuguese following standardized steps according to the recommendations of cross-cultural adaptation guidelines ^16 , 17^ ; 2) validated self-report instruments were employed to assess measures of general and eating-related psychopathology.

We showed that the Brazilian version of the SIG has adequate psychometric properties for assessment of grazing in this sample of undergraduate students. Despite growing research interest in this topic over the last decade, there was a lack of instruments developed for assessment of grazing translated into Brazilian Portuguese. Thus, the present study provides a brief and valid questionnaire that will help researchers and clinicians to evaluate this disordered eating behavior more accurately in the Brazilian context.

Future research should evaluate the reliability and validity of the SIG in larger samples and different contexts, such as clinical and community settings (e.g., people seeking treatment for obesity or ED and in population-based epidemiological studies). In addition, further investigations should explore the associations between grazing subtypes and eating-related psychopathology and thus clarify the role of the LOC as a marker of worse symptomatology.

## Conclusion

In conclusion, the Brazilian version of the SIG demonstrated suitable psychometric properties. Although SIG scores had low stability over time, the instrument showed adequate internal consistency, with both items exhibiting significant associations with related measures. Clinicians need such brief and accurate instruments to help identify this condition in their daily practice.

## Supplementary Material


