# Supplementary material for: Validation of the Brazilian version of the Short Inventory of Grazing (SIG)
Source: Trends Psychiatry Psychother. 2024 Jan 8;46:e20220492. doi: 10.47626/2237-6089-2022-0492 (PMC11140765; doi:10.47626/2237-6089-2022-0492)
Supplement: Supplementary file 1 [file 2238-0019-trends-46-e20220492-suppl.pdf]

http://doi.org/10.47626/2237-6089-2022-0492
Validation of the Brazilian version of the Short Inventory of Grazing (SIG) - de Moraes et al.

Short Inventory of Grazing (SIG)

Autores: Heriseanu AI, Hay P & Touyz S

Tradutores: Appolinario JC, Moraes CEF & Mourilhe C

Nome: ____**Data:**____________

1- Eu gostaria de perguntar sobre o ato de “beliscar” ou “lambiscar”. Com isso quero dizer o ato de **repetidamente** (mais de duas vezes no mesmo período durante o dia) “beliscar” pequenas quantidades de comida **fora das refeições e lanches planejados**.”

NOS ÚLTIMOS TRÊS MESES, tipicamente, quantos episódios desse tipo de “beliscar” você teve por semana?

- ○ 1 – Nenhum
- ○ 2 – Menos de uma vez por semana
- ○ 3 – Uma vez por semana
- ○ 4 – Duas a três vezes por semana
- ○ 5 – Quatro a cinco vezes por semana
- ○ 6 – Seis a sete vezes por semana
- ○ 7 – Oito ou mais vezes por semana
- ○ 8 – Não sabe/não lembra

2- Essa pergunta se refere a quaisquer ocasiões de beliscamento em que **você sentiu que perdeu o controle sobre a sua alimentação** (por exemplo, a sensação de que não poderia evitar voltar a beliscar, mesmo quando tentou "se segurar", ou então quando se sentiu fortemente tentado a beliscar).

NOS ÚLTIMOS TRÊS MESES, tipicamente, quantos episódios desse tipo de “beliscamento” com perda de controle você teve por semana?

- ○ 1 – Nenhum
- ○ 2 – Menos de uma vez por semana
- ○ 3 – Uma vez por semana
- ○ 4 – Duas a três vezes por semana
- ○ 5 – Quatro a cinco vezes por semana
- ○ 6 – Seis a sete vezes por semana
- ○ 7 – Oito ou mais vezes por semana
- ○ 8 – Não sabe/não lembra

Categorias de gravidade

Nenhuma vez ou menos de uma vez por semana	-
1-3 vezes por semana	Leve
4-7 vezes por semana	Moderado
8 ou mais vezes por semana	Grave

http://doi.org/10.47626/2237-6089-2022-0492
